# Digital consultations in Swedish primary health care: a qualitative study of physicians’ job control, demand and support

**DOI:** 10.1186/s12875-020-01321-8

**Published:** 2020-11-24

**Authors:** Hanna Fernemark, Janna Skagerström, Ida Seing, Carin Ericsson, Per Nilsen

**Affiliations:** 1 Primary Health Care Center Lambohov, and Department of Health, Medicine and Caring Sciences, Linköping University, Linköping, Sweden; 2 Centre for Organisational Support and Development, Region Östergötland, and Department of Health, Medicine and Caring Sciences, Linköping University, Linköping, Sweden; 3 Department of Behavioural Sciences and Learning, Linköping University, Linköping, Sweden; 4 Speciality Medicine Centre, Region Östergötland, and Department of Health, Medicine and Caring Sciences, Linköping University, Linköping, Sweden; 5 Department of Health, Medicine and Caring Sciences, Linköping University, Linköping, Sweden

**Keywords:** Digital consultation, Remote work, Primary care, Physicians, Job demand-control support

## Abstract

**Background:**

Digital consultation with primary care physicians via mobile telephone apps has been spreading rapidly in Sweden since 2014. Digital consultation allows remote working because physicians can work from home, outside their traditional primary care environment. Despite the spread of digital consultation in primary care, there is a lack of knowledge concerning how the new service affects physicians’ psychosocial work environment. Previous research has focused primarily on the patients’ point of view and the cost-effectiveness of digital consultation. Hence, there is a paucity of studies from the perspective of physicians, focusing on their psychosocial work environment. The aim of this study was to investigate primary care physicians’ perceived work demands, control over working processes, and social support when providing digital consultation to primary care patients.

**Methods:**

The study has a qualitative design, using semi-structured interviews conducted in Sweden in 2019. We used a purposeful sampling strategy to achieve a heterogeneous sample of physicians who represented a broad spectrum of experiences and perceptions. The interviews were conducted by video meeting, telephone, or a personal meeting, depending on what suited the participant best. The interview questions were informed by the Job Demand-Control-Support (JDCS) model, which was also used as the framework to analyze the data by categorizing the physicians’ perceptions and experiences into the three categories of the model (Demand, Control, Support), in the deductive analysis of the data.

**Results:**

Analysis of the data yielded 9 subcategories, which were mapped onto the 3 categories of the JDCS model. Overall, the participants saw numerous benefits with digital consultations, not only with regard to their own job situation but also for patients and the health care system in general even though they identified some shortcomings and risks with digital care.

**Conclusions:**

This study has demonstrated that physicians perceive working with digital consultation as flexible with a high grade of autonomy and reasonable to low demands. According to the participants, digital consultation is not something you can work with full time if medical skills and abilities are to be maintained and developed.

## Background

Digital consultation with primary care physicians via mobile telephone apps has spread rapidly in Sweden since 2014. The development was initiated by private health care companies, but the service has been taken up by many of the 21 publicly funded regions responsible for providing health care in Sweden. Three of the private companies (Kry, Min Doktor and Doktor.se) have about 90% of the digital consultation market in Sweden [1]. In 2019, approximately 1 million digital consultations with physicians took place in Sweden, representing 5% of all consultation sessions with physicians in primary care. The number of digital consultation sessions increased by 67% in 2019 from the previous year, suggesting a fast expansion in the popularity of this service [1]. Since the start of the coronavirus pandemic, digital consultations with physicians in Sweden have increased by approximately 60% between February and March 2020 for the largest private health companies. The publicly funded health care system also experienced a rapid increase in digital consultations during the same period [2, 3].

The Swedish government has set an ambitious eHealth goal of becoming the best in the world at using the opportunities offered by digitalization to make it easier for people to achieve good and equal health and welfare, and to develop and strengthen their own resources for increased independence and participation in the life of society [4, 5]. The European Commission also aims to achieve more efficient health care by means of eHealth, emphasizing the importance of user-friendly, accessible, and safe care for patients throughout the European Union [6]. The development of the digital health care system can also be explained in relation to numerous developments, such as increased patient involvement in their own health care and higher expectations for access to health care in general [4, 7, 8].

The new digital consultation allows remote working because primary care physicians can work from home, outside their traditional primary care environment [9, 10]. Research on remote working has established that there can be problems concerning social isolation, lack of progress in career opportunities and not being visible in the organization as well as physical disadvantages in the work environment [11, 12]. However, despite the spread of digital consultations in primary care, there is a lack of knowledge concerning how the new service affects physicians’ psychosocial work environment, including their perceived work demands (e.g., perceived time pressure and role conflict between work and family), control over working processes (e.g., decisional latitude and skill discretion), and social support (e.g., interaction with co-workers and supervisors), which has been investigated among remote workers in other populations [13–15]. We have not been able to identify any studies in Sweden or internationally that have investigated how digital consultation is perceived with regard to psychological well-being and psychosocial work environment. Such knowledge is important considering the research demonstrating many problems associated with physicians’ mental health and working conditions [16–19]. Studies in many countries in Europe and the United States have shown high and increasing perceived stress and burnout among physicians [16–20]. Furthermore, primary care physicians’ workload seems to have increased due to an increase in demand for health care among citizens as well as an increase in bureaucratic paperwork and tasks viewed as illegitimate by physicians [7, 20–22].

Previous research on digital consultation with physicians has primarily focused on the patients’ point of view and experiences of such meetings [23–27]. Studies have also investigated the cost-effectiveness of digital consultation [28, 29]. Hence, there is a paucity of studies concerning digital patient consultation conducted from the perspective of physicians and focusing on their psychosocial work environment. Addressing an important knowledge gap, the aim of this study was to investigate primary care physicians’ perceived work demands, control over working processes, and social support when providing digital consultation to primary care patients.

## Methods

### Study design and setting

The study has a qualitative design, using semi-structured, individual interviews. A qualitative approach was chosen because little is known about the psychosocial working conditions of primary care physicians in digital consultations with patients. Individual interviews were considered the most relevant method for gaining a deeper understanding of the physicians’ working conditions allowing participants to speak freely and not be affected by other participants’ experiences and opinions of the topic.

The Swedish health care system consists of 21 regions (previously known as county councils), which provide health care for the population, mainly funded by taxes. All residents are insured by the state with equal access to health care for the whole population. Fees are low and regulated by law. Primary care (both traditional and digital consultation) is provided by both public and private actors, with the private providers representing 43% of the total number of primary care units in Sweden 2018 [28]. Private health care companies dominate the market with regard to digital health care services (90%), but regions are increasingly launching their own digital consultation services [1, 30, 31]. The private health care companies included in our study are contracted to regions; the out-of-pocket fees for their patients are equal to that of publicly funded health care [32].

### Recruitment of participants

We used a purposeful sampling strategy to achieve a heterogeneous sample [33] with regard to (1) involving physicians working with digital consultation in both publicly funded primary care units and in private health companies; (2) the geographic location; (3) different experiences of working as a physician, i.e., both specialists and residents. The objective of this sampling strategy was to recruit physicians who represented a broad spectrum of experiences and perceptions.

To recruit participants working in public care, we contacted all 21 regions in Sweden by examining the regions’ websites to identify the person who seemed to be responsible for digital consultation in the region. We sent an e-mail to this person, briefly informing them about our study and asking for physicians from the region to participate. We did not receive any response from 8 regions, whereas 4 regions agreed to participate and provided contact information to key persons in the organization to enable us to establish contact with physicians who had worked with digital consultation. The remaining 9 regions responded that they did not offer digital consultation and/or declined participation. We approached 29 primary care physicians from the 4 regions, of which 17 agreed to participate.

To recruit participants working in private health care, we approached 7 private companies. Of these, 5 agreed to participate in our study. We approached 12 physicians from these companies, of which 11 agreed to participate. All the participants had some experience of employment in publicly funded health care. Thus, in total we recruited 28 physicians for the study (Table 1).

**Table 1:** Participant characteristics

Characteristic	Number (%)
Sex	
Male	12 (43)
Female	16 (57)
Age	
28–39 years	10 (36)
40–49 years	10 (36)
50–63 years	8 (28)
Level of medical training	
Specialist in primary care medicine	18 (64)
Resident in primary care medicine^a^	8 (28)
Specialist in other specialty than primary care	1 (4)
Not specialist or resident	1 (4)
Employer	
Publicly funded health care	17 (61)
Private company	11 (39)
Type of work	
Combined digital work with traditional consultation	25 (89)
Digital work exclusively	3 (11)

^a^In Sweden, resident physicians have finished medical school, possess medical license and for 5 years they both provide healthcare and are learning to become specialist physicians (in this case, specialists in primary care medicine)

Before the interviews were conducted, the participants signed informed consent stating that their confidentiality was guaranteed and that no one other than the interviewer would know their identity. To the other researchers, the participant was known only by initials and other demographic, non-identifying data. No participant withdrew participation during or after the interviews.

The study was approved by the Ethics Review Board in Region Östergötland (2019–01910). Transcripts are stored in the authors’ password-protected computers with no access for anyone other than the authors.

### Data collection

The authors developed a semi-structured interview guide to capture the physicians’ perceptions and experiences concerning the psychosocial work environment of digital consultation. The interview guide was assembled by the research team behind the study, based on the existing literature on psychosocial work environment [14].

We pilot tested the interview guide in 2 interviews, which indicated that further questions regarding the digital work environment needed to be incorporated into the interview guide. Despite this, the first 2 interviews included relevant information and were therefore included in the analysis.

The interviews were conducted by all authors except PN and JS. Each interview lasted between 24 and 84 min and was digitally audio recorded. No field notes were taken during or after the interviews. The interviews were conducted in by video meeting, telephone, or a personal meeting, depending on what suited the participant best. During the interviews, only the participant and interviewer were present to allow the participant to speak freely. Except for the 2 participants in the pilot interviews and additionally one participant who was known to HF, none of the participants had any relationship with the researchers. The first 3 interviews were transcribed verbatim by HF and the remaining interviews were transcribed by a professional transcription agency. All transcripts were carefully examined by HF to ensure accuracy. The interviews took place between April and October 2019. Saturation of data was discussed by the research team, and according to earlier research, the major themes and codes are derived from data after 12 interviews. This gives our study with 28 participants a convincing strength.

### Theoretical framework

The interview questions were informed by the Job Demand-Control-Support (JDCS) model [14, 34]. The model was also used as a framework to analyze the data by means of categorizing the physicians’ perceptions and experiences concerning digital consultation into the 3 categories of the model, i.e., Demand, Control, and Support, in the deductive analysis of the data.

The Job-Demands model was originally developed by Karasek [35]. Job demands may involve time pressures and role conflicts, whereas job control refers to employees’ ability to control their work situation [13, 14]. Combining the 2 dimensions of job demands and job control, Karasek [14] stated that jobs high on demands and low on control (“high strain jobs”) carry a high risk of development of adverse psychological symptoms such as anxiety and depression, but also cardiovascular disease [34]. By contrast, in jobs that are low on demands and high on control (“low-strain jobs”), adverse reactions are unlikely.

The Job-Demands model was later extended by Johnson and Hall [34] who added the dimension of Support at the workplace as a third dimension. The support dimension was introduced to represent a “buffering” effect on perceived stress, mental health, and job strain by moderating the negative impact of high strain. The JDCS model predicts that work situations characterized by high demands, low control, and low social support are the most harmful for workers’ well-being [34]. The JDCS model has been used worldwide in research on psychosocial work environment and occupational stress [13, 14, 34, 36].

### Data analysis

Participants’ responses were analyzed using directed content analysis according to Hsieh and Shannon [37]. As a first step, all authors read all transcripts to obtain an understanding of the whole. HF then coded the transcripts by identifying key concepts as initial coding categories. Thereafter, HF and JS met several times and discussed the coding process as well as developed tentative operational definitions for each category, in this case according to the JDCS model. Then, all the authors read and discussed the findings. After discussion in the group there were some suggestions regarding reorganization of some of the categories.Eventually, consensus was reached on the categories and labels for the categories. Representative quotations from participants were proposed by HF and were discussed with the rest of the team before the final quotations were agreed upon. Quotations are marked from physician #1 to physician #28 in the Results.

## Results

The 28 participants displayed different characteristics (Table 1). Seventeen were employed by publicly funded primary care units in 4 regions and 11 worked in 4 different private companies. Of the 4 private companies, 3 work predominantly with digital consultation although they also offer traditional consultation to a limited extent, and the fourth company offers digital consultation as an option besides their regular primary care. The public regions offered digital consultations as a minor part of their regular primary care. The publicly employed physicians either performed digital consultations as part of their regular employment or as an additional, reimbursed task. Participating regions and companies were located in urban areas in central and southern Sweden. However, the patients could be expected to live in both rural and urban areas since the service is available to patients from the whole of Sweden, i.e. without geographical restrictions. Thirteen of the participants had received their medical training in Sweden, and 15 had undergone medical education abroad. Digital consultation was carried out both during office hours as well as in the evenings and at weekends.

The analysis of the data yielded 9 subcategories, which were mapped onto the 3 categories of the JDCS model (Table 2). The results of the analysis for each category and subcategory are presented in the following sections.

**Table 2:** Categories and subcategories

Control over job situation	Demands of job situation	Social support in job situation
Autonomy	Workload	Colleagues
Competence	Patient safety	Supervisors
Technology	Resource use	Patients

### Control over job situation

Control over job situation concerns employees’ ability to organize their work, adopt their own initiatives, and have latitude about decisions. This category consists of 3 subcategories: autonomy, competence, and technology.

#### Autonomy

Participants experienced a great deal of decisional latitude concerning their work situation. The digital consultation allowed for high levels of flexibility in terms of deciding working hours and choosing where to work, including having the ability to work from home. Several participants saw the opportunity to determine the location of work as very positive. They stated that they had more time to spare and could spend valuable time with their family when they did not have to travel to and from work. However, some shortcomings were also expressed, e.g., not having scheduled coffee breaks and uncertainty about whether they could take breaks as usual or not.

You’re at home. The advantage of this [digital consultation] is that I don’t have to travel to and from work. I start at 8 a.m. and I sit down at my computer at 8 and when I quit at 5 p.m. I’m already at home. It’s an incredible benefit. [#24]

#### Competence

Concern regarding loss of clinical competence when working exclusively with digital consultation was expressed by participants. They underscored the importance of combining traditional clinical consultation and digital consultation to maintain and develop their medical proficiency. There was agreeance among the participants as regards the concern about the lack of improvement in their medical skills because they experienced that digital consultation consisted mostly of relatively simple medical issues. Lower urinary tract infections in women, upper airway infections, allergies, prescription renewals, and certification of sick leave were common issues.

Digital consultation, that’s something else, it’s an easier approach to work. It’s some sort of symptomatic treatment for, most commonly, one condition, which means you can close the case and the patient is content, and you’re pleased because there’s no more work to do when finishing the case. To work only with digital consultation, I don’t think that’s a solution because I can’t imagine you’re able to maintain your competence if you only work digitally. [#6]

#### Technology

The technology used in digital consultations caused participants some problems, including recurring issues with the internet connection. This was experienced as particularly worrisome when the connection with the patient was lost in the middle of a consultation and the physician was not able to reach the patient again. Further, participants reported that there were some disadvantages working from home because they lacked technical support when working outside office hours, forcing them to try to solve technical problems by themselves. However, most of the participants experienced very good technical quality, e.g., concerning pictures of skin conditions. Several of the participants saw potential in incorporating the digital consultation system into their own everyday journal system rather than using a different system for digital consultation.

Yes, the technical challenge is that we’re not primarily working in our regular computer system but in another system. For this to be really efficient, it needs to be integrated into our existing journal system. So, there are technical parts that can be improved. [#28]

### Demands of job situation

Demands of job situation are the physical, psychological, social, or organizational aspects of jobs that require physical and/or psychological effort. Perceived time pressure and role conflict (incompatible demands related to one’s job or position) are also a part of the demand dimension. Three subcategories were identified in this category.

#### Workload

Participants experienced that the workload was significantly lower when working with digital consultation. They explained this in terms of non-existing production demands, i.e. number of consultations with patients per hour, in combination with the type of medical cases they handled in digital care. However, participants employed by private companies had seen an increase in demands of production, e.g., the number of patients they were expected to counsel per hour. The privately employed participants described that they had a very reasonable workload from the outset, with no demands that they must administer a certain number of patients per hour, but they noted that this was changing as patient volumes increased, which they perceived to be a shortcoming. The increased demands had led some of the participants to look for other employers who could offer more favorable working conditions.

The workload was quite low, the patient cases were usually very simple to solve in 15 minutes, so there were no large problems. You managed that without any problems. [#3]

#### Patient safety

The participants believed maintenance of patient safety when working with digital consultation was part of the job demands. They noted that digital consultation had some shortcomings that could have a negative impact on patient safety, e.g., when patients sought medical advice for conditions that were not suitable for digital consultation or when there were technical problems so that the contact with the patient was lost and the patient could not be reached again. Some participants stated that difficulties in mutual understanding between physician and patient was quite common when there were language differences. This communication problem could negatively affect patient safety, according to some participants.

For I think, for example, sometimes when your patients’ Swedish [language] is very poor, I feel, and I actually think many agrees with me, that it is better with a traditional consultation where you really can sort things out and talk, face to face. [#17]

Another concern about patient safety expressed by the participants was the lack of a unified documentation system. It was viewed as risky to prescribe medication without knowing what other medications the patients were prescribed since a majority of patients were unknown to the physicians when working with digital consultations.

#### Resource use

Digital consultation requires that the participants handle medical cases suitable for digital care. The participants raised concerns about the risk of generating “unnecessary” health care with digital consultation, making them somewhat ambivalent towards the use of their medical competence in the digital consultations. They noted that the digital consultation predominantly reached young, presumably healthy individuals, possibly at the expense of the elderly population with a higher burden of disease. Issues concerning the use of constrained health care resources created concern for some of the participants. The question about who decides what is, and what is not, an important medical condition was also brought up. Participants perceived that some patients used digital consultation as a form of health care advice service, which they considered as unnecessary health care because a free health care advice service already exists in Sweden. In most regions and private companies represented in this study, digital consultation means that patients first get in touch with highest medical level (i.e., a primary care physician) instead of, e.g., discussing their problems with a nurse as a first step. Some participants expressed that they would like to see some sort of selection of patients because a large proportion of patients did not need to see a physician if limited health care resources were to be used most effectively.

In my opinion, health care today is very, very available for quite young, healthy individuals. But for those who are really the most ill, it can be a complicated system and difficult to access. [#15]

Moreover, it [digital consultation] may be a waste of resources, like I told you before, you immediately get the highest level of competence, in a very convenient and available way. And that’s really the greatest risk, that you pay a lot for a small measure from the health care system. [#3]

### Social support of the job situation

The social support of the job situation includes helpful interactions with colleagues and supervisors. Social contacts and structures affect basic psychologic mechanisms, which are important for well-being in the long term. Social support can also influence job stressors in a positive direction, acting as a “buffering” mechanism.

#### Colleagues

Participants conveyed that they could feel somewhat socially isolated when working with digital consultation from home and/or in the workplace outside office hours, which generated uncomfortable feelings of loneliness. This was mostly reported by participants in publicly funded health care who were accustomed to having a regular workplace to go to. Participants employed by private companies did not express the same sense of loneliness or isolation. On the contrary, they had the opportunity to interact with remote colleagues through internal chat forums where they could obtain both medical advice and support from others.

We have this community, it’s a fantastic source of support among colleagues concerning both work environment issues and also practical concerns, but also concerning knowledge about patients. So it’s really … It’s invaluable. It is not possible in regular health care to have that sort of contact with colleagues. Here, we have that possibility and you can also receive a lot of support from your colleagues and the employer too. But this collegial interaction with colleagues is so valuable, so valuable. [#20]

With regard to support from colleagues in health care in general, i.e., not necessarily colleagues in digital consultation, most participants reported non-supportive attitudes overall. This lack of collegial support seemed to generate feelings of shame and guilt among some of the participants, with some even avoiding telling colleagues about their work as a “digital doctor” to avoid being taunted.

You feel ashamed, you know how others react to this “so you’re after the easy money, how bad you are, taking it from taxes”. They mean the cheapest health care goes to traditional primary care and that we (the digital physicians) take the easy patients from traditional primary care, or “unnecessary” patients. Many people think like this, which isn’t true. Most of the patients haven’t been able to get help from traditional primary care. [#18]

#### Supervisors

Some participants employed by private companies reported an obvious lack of support from supervisors, i.e., health care colleagues in management positions. There had initially been regular staff follow-ups, but they believed that they were no longer able to discuss working conditions with their supervisors. Participants working in publicly funded health care experienced supervisor support differently. In most cases, they perceived satisfactory support from primary care supervisors. However, some of the publicly employed participants expressed that their supervisors were skeptical of digital consultation and did not encourage them to work with this way, which left them feeling unsupported and somewhat alone.

When it comes to work environment, most of it is ok I figure, but a great deal has worsened. For me, I run my cases, but I want my work to be acknowledged as well. From patients I usually get acknowledged but from my employer it has become worse. In my opinion, the employer should be more attentive regarding their employees. This is even more important when employees are doing teleworking and not sitting in a physical office. Initially, the employer was very good in acknowledging my work, but not anymore, so that’s a disadvantage. [#25]

#### Patients

Participants experienced considerable patient support for the digital consultation. Many patients were very positive and grateful for the fast, readily available care they received, which they often communicated directly to the participants. This response generated feelings of satisfaction among the participants, who felt they could really help patients solve their problems.

I told you before, I’ve never had patients this pleased/content, one after another, everyone’s happy with your work. Ok, it’s not the most complex burden of diseases, but still, the patient’s in need of a solution for a symptom that bothers them. You can solve it easily, readily for all, for you. I think everyone’s very happy with this way of solving the problem. [#6]

However, some of the participants also observed that digital consultation situations could create a more anonymous form of physician-patient communication, making some patients lose their inhibitions so that they were occasionally quite rude and sometimes said inappropriate things they probably would not have done in a face-to-face consultation.

## Discussion

The aim of this study was to investigate primary care physicians’ perceived work demands, control over working processes, and social support when providing digital consultation to primary care patients. We found that physicians working with digital consultation experienced several advantages with this type of work. The physicians perceived that the job demands were reasonable because the medical cases handled in digital consultation were generally not too complicated. Moreover, the physicians experienced a high level of autonomy and perceived control over their work, which they considered to be very valuable. The physicians also felt they received a great deal of social support from patients, which they found very satisfying. Overall, the participants saw numerous benefits, not only with regard to their own job situation but also for patients and the health care system in general, even though they identified some shortcomings and risks with digital care. Some of the perceived shortcomings with digital consultations were technical issues, concerns regarding patient safety and lack of development in physicians’ medical skills if working exclusively with digital consultations.

Concerning job control, the participants seemed to value the flexibility and autonomy of digital consultation very highly. The ability to choose where and when to work was a considerable advantage with digital consultation over traditional primary care work. Participants felt they had more time to spend with family when they could work from home. These findings are consistent with earlier research concerning remote working, which has shown that flexible working hours and schedules have a positive effect on productivity and outcomes, such as increased job satisfaction, lower turnover intentions, and reduced role stress (i.e., combining professional role with family role) [15, 38]. This finding suggests that there could be some shortcomings with regard to these particular factors in traditional primary care and that physicians could benefit from more control over their working situation, e.g. when and where to work.

The freedom of being able to choose when and where to work was highly appreciated by the participants. They do not have this sort of autonomy in the traditional primary care setting, which largely lacks opportunities for employees to have a flexible working schedule [39]. One way of increasing flexibility for primary care employees could be to integrate digital consultation into the schedule of the daily work in traditional primary care, as well as employers offering the opportunity for physicians to work elsewhere (i.e., at home or other out-of-office arrangement). This would give physicians more decisional latitude on how and when to meet their patients.

The participants identified the technical equipment used for digital consultation as an occasional source of problems. The participants could not control these aspects of the consultation, but it occasionally caused them problems; patients were “lost” and could not be reached again after internet connection issues. Similar technical issues have been reported as a shortcoming and a source of stress in other studies concerning digital consultations [8, 40, 41]. Technical issues and problems were more common and pronounced among participants employed by publicly funded health care compared with participants employed by private companies, who perceived the technical equipment as well developed and highly user friendly. The technical shortcomings were also raised as a potential risk regarding patient safety. If the physician could not reach the patient after connection was lost it could generate anxiety or stress for the physician depending on the medical condition of the patient. This type of potential problem would contradict the goal of health and medical care according to the Swedish law of healthcare that good health and care on equal terms should be provided for the entire population [42].

The participants raised some concerns regarding the potential misuse of health care because the digital route to medical advice is very convenient for patients. This, in turn, generated thoughts about who is entitled to health care and to what extent health care is available for everyone. Similar questions and opinions have been raised by both clinicians and researchers and caused a lively debate in newspapers and medical papers in Sweden as well as in the United Kingdom and in the United States [22, 43, 44]. On the other hand, participants discussed potential unloading of traditional care when patients with minor health problems turned to digital consultations instead of seeking physical care in a traditional way. The participants emphasized that this could possibly lead to more time available for other patients with multiple, chronic diseases and for the elderly. This potential unloading has not been realized in practice; instead, these studies suggest that there is an increase in the number of patients seeking medical advice digitally [22, 43, 45].

Regarding social support when working with digital consultation, the participants expressed a feeling of satisfaction in that the patients were very rewarding. Some of the participants felt somewhat socially isolated and lonely from time to time. However, others thought the interaction with colleagues improved compared with traditional primary care consultation in that they could get in touch with colleagues through various electronic channels and chat forums. This interaction provided an opportunity to learn from other physicians who were also engaged in digital consultation. Digital consultation enabled the physicians to work from home, i.e., remote working. Earlier research on this type of work has shown that remote working does not have to impede employees’ social contacts with co-workers [38]. However, the findings are inconclusive regarding social support and its effects on psychologic well-being and job satisfaction for employees who engage in remote working in general [12, 46, 47]. Vander Elst et al. [12] showed that remote workers experienced lower social support from colleagues the more extensively they worked from home and that this was related to higher levels of adverse psychologic symptoms, such as cognitive stress and emotional exhaustion. This finding is congruent with the results in our study because several of the participants expressed that remote working was something they did on a part time basis. The participants employed by private companies experienced slightly more support from co-workers than publicly employed participants. This could possibly be explained by the well-established digital chat forums available for the employees as well as the opportunity to work from the common office rather than from home whenever they chose.

There were some differences in perceived support from supervisors between the physicians in our study who were employed by publicly funded health care and those who were employed by private companies. These differences entailed, among other things, a decline in support from supervisors in private companies compared with the situation at the start. The participants in publicly funded health care received sufficient support from supervisors although there were some differences in supervisors’ enthusiasm for digital consultation, which could affect employers’ experience of support in a negative way. Digital working is a change in the physicians’ way of working and seeing patients, therefore it is important to have supervisors’ support because earlier research has shown that this can mitigate change-related stressors, such as exhaustion and cynicism among employees [46].

Another notable finding in our study was the satisfaction most of the participants expressed with patients who conveyed their appreciation and gratefulness to the physicians with the consultation. This sort of recognition was highly rewarding and somewhat of a new experience for the participants, who were not used with this type of direct and positive feedback from patients. The patients were satisfied with receiving help almost instantly when seeking digital care, and this made them grateful to the responsible physician. Patients actually expressed amazement with how quick the service was, according to the physicians. The participants were frequently told that this contrasted with many patients’ earlier experiences with traditional primary care. Research on the patient-doctor relationship in the digital era suggests the need for physicians to acquire new skills in the consultation to be able to diagnose patients without a physical examination as well as interact through digital channels with patients they may not have met before [48]. Furthermore, Mesko and Győrffy [48] propose a change in the patient-doctor relationship to more of a partnership. The findings in our study are also in line with earlier research concerning communication and the relationship between caregiver and patient; patients may experience the digital way less intimidating and find it easier to say what is on their mind than in traditional consultations [49].

Our findings suggest that digital consultation entails lower job demands than traditional work as a physician in primary care. The participants observed that they predominantly handled easier medical cases in digital consultations. The combination of high levels of control and low demands is well established in low-strain jobs, according to Karasek and Theorell [14]. This type of job yields few, if any, challenges for employees, but in return stress levels are low and the risk of experiencing adverse strain is generally very low, which makes this kind of job highly desirable. Earlier research has shown that being a physician is an occupation with high demands and medium-to-high control, which leads to the development of competence and increased learning and motivation (e.g., active job), which the low-strain job does not [14]. This lack of development and learning was also a concern raised by several of the participants in our study. There seemed to be consensus among the participants that they did not want to work exclusively with digital consultation; they wanted to continue with traditional primary care work as well to maintain and develop their competence. In the short term, digital consultation seemed to provide some relief for stressed physicians, but in the long term they believed this work could disqualify them from working in regular health care.

This study has a few limitations that should be noted when interpreting the findings. We chose a qualitative approach because little is known about primary care physicians’ views on digital consultation. For this reason, we conducted interviews with physicians to gain a deeper understanding of the topic. Participation was voluntary; the interviewees were included in the study after they actively expressed their interest in participation. This means that the participants may have been particularly interested in the topic and/or early adopters with a curious and positive mindset. The findings of the study cannot be directly transferred to international settings. The transferability of our results is limited to primary care settings in Sweden, although participants were recruited from different types of employers and forms of employment. Despite this, the results in our study may be applicable to other settings because the sample is considered variable and adequate [50]. It would have been interesting to interview physicians in more rural parts of Sweden but by the time of this study, not all regions in Sweden offered patients digital consultations with primary care physicians. However, the private companies offer their service to patients across Sweden and the public companies have broad uptake areas which is why the patients consulting digital doctors could be expected to live in both rural and urban areas. Instead of statistical generalization, we sought analytical (theoretical) generalization by comparing findings with comparable research to the extent it was possible due to the limited amount of research on physicians’ digital consultation in primary care.

The study also has considerable strengths. The multidisciplinary research team enhanced the credibility of the study, because it allowed different perspectives on the issue under study [51]. The team consisted of the following professions: physician (HF), behavioral economist (PN), political scientist (IS), public health researcher (JS), behavioral scientist (CE). Another strength was the relatively high number of interviews (*n* = 28), although Malterud et al. [50] have emphasized that the strength of the information received (information power) is more important than the size of the sample. Regardless, this enabled us to use quotations from many different participants, adding transparency and trustworthiness to the findings. It was also a strength that the participants came from different geographic regions of Sweden and from both public and private organizations. Another strength was that the participants included men and women of different ages and with different experiences from previous primary care work.

Implications for further research in the area are important because the work with digital consultation differs greatly from the traditional consultation approach that is part of physicians’ education. Digital consultation is developing rapidly and further research on how it affects physicians in primary care and other settings is important. According to the findings in our study, digital consultation could be one way to increase physicians’ perceived autonomy and reduce stress levels to some extent if it is incorporated in the daily or weekly work. This could possibly lead to increased job satisfaction and reduced burnout among physicians, which is an important issue. It is also important that digital consultations are combined with traditional consultation so that competence is maintained and developed.

## Conclusion

In conclusion, this study has demonstrated that physicians working with digital consultation perceive their work as flexible with a high grade of autonomy. The demands of digital work are reasonable to low. According to the participants in our study, digital consultation is preferably not something you can do full time if medical skills and abilities are to be maintained and developed. Participants’ perceptions of social support are that it is pronounced among patients and to a lesser extent from colleagues and supervisors.

Digital patient consultations have increased in the last few years [1], a development which has intensified tremendously due to the outbreak of COVID-19. Even so, there is an obvious lack of research concerning physicians’ occupational health, psychological well-being, and job satisfaction in telework, which constitutes a new way of working for physicians. Further research in this area is important, partly because it is a rather new phenomenon but also because there are so many reports concerning physicians’ mental health and occupational stress.

## Supplementary Information

**Additional file 1.** Interview guide - Digital consultations with physicians.

## Data Availability

For access to the data used in the current paper, please email hanna.fernemark@liu.se. All interview data are in Swedish language.

## Abbreviation

JDCS: Job Demand-Control-Support
