# Supplementary material for: Digital consultations in Swedish primary health care: a qualitative study of physicians’ job control, demand and support
Source: BMC Fam Pract. 2020 Nov 24;21:241. doi: 10.1186/s12875-020-01321-8 (PMC7684852; doi:10.1186/s12875-020-01321-8)
Supplement: Supplementary file 1 — Additional file 1. Interview guide - Digital consultations with physicians. [file 12875_2020_1321_MOESM1_ESM.docx]

**Interview guide - Digital consultations with physicians**

*This project concerns digital physician-patient consultations from the perspective of the physicians’ work environment conditions. The focus of the interviews will be on your experiences and thoughts concerning this type of work.*

**Work**

*Oral instruction*

*First, I would like to talk about your work, as a* *doctor,* *in more general terms, before we address the work and work environment conditions regarding your regular workplace. Finally, we will discuss the work and work environment conditions when carrying out digital patient consultations.*

How come you chose general medicine as a specialty?

For how long have you worked at your current workplace?

What is the most important thing for you in your profession as a doctor?

What is the best thing about your work?

What conditions should be fulfilled to make you feel like you are able to do a good job?

How do you experience the balance between private and working life?

**Work environment conditions - regular workplace**

How do you appreciate your work situation and workplace overall?

Can you describe a normal working day for you?

How do you experience the work conditions at the primary health center?

- Work environment (mental and physical)?
- Workload (overtime)?
- Are there areas in your work you would like to spend more or less time on?
- How do you perceive the expectations on you, from management, colleagues, patients?

Have the work environment conditions changed during your time in the workplace?

How flexible do you feel that your work is regarding time management? Would you like it to be different – how?

How do you experience support for your work from management and/or manager?

How is the collegial support and cooperation among the doctors and concerning other professions at your workplace?

- Examples of collaborations that have turned out well?
- Examples of collaborations that have turned less favorably

**Digital consultations**

During which period did you work with digital consultations or are you currently working with digital consultations?

What do you think about digital physician-patient consultations in general?

How come you decided to work with digital consultations?

How did you experience the planning/organization/implementation of digital consultations?

What expectations did you have before starting to work with digital consultations?

How did you experience the preparation for working with digital consultations?

- Training?
- Technical equipment?

Is there cooperation between the digital health centre (or similar) and your regular workplace?

- Management’s reaction to your interest in digital consultations?
- Opportunities to work during the day/evening?

Describe how a regular work shift might be like when working with digital consultations?

- Length of work shift?
- Distribution of shifts?
- Co-operation or with colleagues?
- Location and ergonomics?
- Other factors influencing the consultation *(internet connection, technology, local, privacy)*?

How flexible do you believe that your work with digital consultations is in terms of time management/tasks?

How do you experience the work situation when working with digital consultations regarding:

- Work environment (mental and physical)?
- Workload?
- Expectations of you?
- Areas you want to spend more/less time on?

How does the work with digital consultations differ from the regular work at your health center?

What challenges, difficulties or risks have you identified when working with digital consultations? (*Tell me about a situation where it was difficult. What made it difficult? Could it have been done otherwise?)*

What do you think about your continued work with digital consultations in the future?

**Concluding questions**

What benefits do you see with regular patient meetings at a clinic and with digital consultations, respectively?

Are there disadvantages to each form of patient meetings (regular vs. digital), if so, which?

What attitudes towards digital consultations with physicians have you encountered? *(Colleagues, co-workers, family and patients?)*

What, in your opinion, are the main challenges of digital consultations with physicians?

What, in your opinion, are the main challenges for primary care, in general, in recruiting and retaining staff?

Is there something I have not asked about that you think I should have addressed – if so, what?

Is there anything else you would like to add before we finish the interview?

If not, I would like to conclude with some brief personal background questions:

**Personal background**

Age

Sex

Native language

City of education/country

When did you receive your medical license?

Specialist or Resident (for how long specialist/how long as resident)

Additional specialist skills?

Further training?

Marital status? Children under the age of 15 in the household?

Are you employed by publicly funded healthcare or private healthcare provider?

Full/part-time? (Justification)

Side tasks? Internal/external?

Thank you for your participation!
